# Fit for fight – self-reported health in military women: a cross-sectional study

**DOI:** 10.1186/s12905-019-0820-4

**Published:** 2019-10-17

**Authors:** Elin Anita Fadum, Leif Åge Strand, Monica Martinussen, Laila Breidvik, Nina Isaksen, Einar Borud

**Affiliations:** 1 Institute of Military Medicine and Epidemiology, Norwegian Armed Forces Joint Medical Services, B28A N-2058, Sessvollmoen, Norway; 2 RKBU North, UiT The Arctic University of Norway, Tromsø, Norway; 3 The Norwegian Defence University College, Oslo, Norway; 4 UiT The Arctic University of Norway, Tromsø, Norway

**Keywords:** Norway, Self-reported health, Military, Female, Surveys

## Abstract

**Background:**

Substantial research has found that women assess their health as poor relative to men, but the reasons for this are not fully understood. Military women are characterised by good health and the ability to work in an archetypically male culture. Thus, studies on the gender pattern of self-reported health in military personnel could generate hypotheses for future research on the possible associations between gender and health. However, such studies are rare and limited to a few countries. The aim of this study was to examine self-reported physical and mental health in Norwegian military women.

**Methods:**

We compared responses on self-reported health of 1068 active duty military women in Norway to those of active duty military men (*n* = 8100). Further, we compared the military women to civilian women working in the Norwegian Armed Forces (*n* = 1081). Participants were stratified into three age groups: 20–29; 30–39; and 40–60 years. We used Pearson Chi-square tests, Students t-tests and regression models to assess differences between the groups.

**Results:**

The military women in our study reported physical illness and injuries equal to those of military men, but more military women used pain relieving and psychotropic drugs. More military women aged 20–29 and 30–39 years reported mental health issues than military men of the same age. In the age group 30–39 years, twice as many military women assessed their health as poor compared to military men. In the age group 40–60 years, more military women than men reported musculoskeletal pain. Military women used less smokeless tobacco than military men, but there were few differences in alcohol consumption and smoking. Military women appeared to be more physically healthy than civilian women, but we found few differences in mental health between these two groups.

**Conclusion:**

Most military women reported physical symptoms equal to those of military men, but there were differences between the genders in mental health and drug use. More favourable health compared to civilian women was most evident in the youngest age group and did not apply to mental health.

## Background

Substantial research has found that women and men who participate in surveys assess their health quite differently. Female subjects tend to report more physical and mental health problems than men and more often perceive their health as poor [1]. Contrary to this, women have more healthy lifestyles [2]. Among the few health-maintaining behaviours men perform more often than women is heavy physical activity (PA) [3], and compared to women, men rarely ingest harmful doses of drugs [4].

The exact mechanisms behind this pattern remain unclear, but some of the most common explanations are based on sex-determined biological factors, gender differences in reporting behaviour, and differences in male and female psychology regarding things like risk perception, illness definition, and coping strategies [2]. The pattern is further complicated because the direction and magnitude of gender differences in self-reported health vary according to countries, cultures, context and age [5–7]. Therefore, research has focused on whether inequalities in living and working conditions are related to gender differences in health problems [8]. Some claim that the segregation of women into sedentary or repetitive work, or to positions with lower pay and lower status, could be explanatory factors for their disadvantageous health [9, 10]. However, findings from the Scandinavian countries challenge such assumptions, as these countries do not exhibit gender equality in self-reported health, despite their characterisation as the most progressive countries in terms of gender equality, and their generous universal welfare systems [6, 11].

Military women are carefully selected and extensively trained based on their good physical health, and mental robustness. Military women are required to integrate a typically masculine institution; they must cope with stress and function physically on the same level as their male military colleagues. The Armed Forces performance expectations and wages are basically gender-neutral. Therefore, one could expect that military women would consider their health to be the same as that of military men, and superior to that of most women in the general population [12]. However, although the military fosters good health, it is conceivable that being a woman in the military is associated with significant physical and psychosocial stressors and the adoption of typically “male” unhealthy behaviours, such as harmful drinking and increased tobacco use [13–15]. If so, military women could be at a higher risk of compromising their health the longer they stay in the military [16, 17]. One can speculate on whether military women’s self-reported health remains superior or becomes more equal to that of civilian women as they grow older [18].

Previous research has found gender equality in self-reported health among military personnel [19, 20], but there are also studies indicating that military women report less favourable mental and physical health outcomes compared to military men [21, 22]. One study found that active duty military women in the US Army reported better physical health than civilian women [17]. Surveys conducted in European countries did not demonstrate such differences, and it is unclear how military women assess their mental health relative to civilians [20, 23]. However, few studies have examined self-reported health in military women early and late in their careers and there is a gap in the literature on self-reported health in military women serving in northern Europe. Studies on how military women of different ages assess their health relative to their counterparts among military men and civilian women are important in military and occupational epidemiology, as they may aid in the generation of hypotheses about the complex interplay between gender, working environments, and health.

## Methods

### Aim

The aim of this study was to examine self-reported physical and mental health in Norwegian military women. Based on previous research, we assumed that military women’s assessment of their health would be equal to the assessment of military men and better than the assessment of civilian women employed by the Armed Forces [10, 12, 20]. We wanted to explore if the expected differences would be present in different age groups and examine if increasing age had a different impact on self-reported health in the three groups of military women, military men and civilian women.

### Setting and participants

The Norwegian Armed Forces Joint Medical Services conduct a health survey among military and civilian employees of the Armed Forces every other year. All personnel employed on 1 January of the year of the survey receive an invitation to participate via e-mail with a web-link to the survey. Data collection goes on for 6 weeks. Those who do not respond after the first invitation receive one to three reminders, and a notification 24 h before the survey closes. Responses are linked to the unique number that is assigned to each Norwegian Armed Forces employee and are stored as the individual’s personal information in the Norwegian Armed Forces Health Registry.

The current study has a cross sectional design utilizing survey data from 2015 and 2017. To maximize statistical power, we combined the two datasets to one study sample. If participants took part in both surveys, we only used their responses from 2017. A total of 18,947 unique individuals were invited to participate in one or both of these surveys, and 12,903 (68.1%) completed. Preliminary analyses revealed that all the military women who participated were ≤ 60 years of age. Therefore, 192 military men and civilian women aged > 60 years were excluded, in addition to all civilian men (*n* = 2462). The final analytical sample included 10,249 military women, military men, and civilian women.

### Measures

Information on the participant’s military status, sex, and birth year was retrieved from the Armed Forces’ personnel administrative database. *Military women and military men* include officers of any rank and grenadiers. *Civilian women* include women who were employed by the Norwegian Armed Forces in positions that do not require military training. Civilians may work in the Armed forces in a range of positions i.e. academic positions, engineering, human resources, law, economics etc. Civilian employees are not required to have passed military training, to have a military health certificate, or to wear a military uniform at work. Their education, training and salaries varies with their position.

Information on health was obtained from the survey, which was designed by the Norwegian Armed Forces Joint Medical Services and calibrated to the largest epidemiological surveys in Norway (Cohort of Norway) [24].

#### Physical health

The first survey question was: *How is your current health status? (Response options: poor/not so good/good/very good)*. Those who responded poor/not so good were coded as having ‘poor health’; those who responded good/very good were used as the reference group.

Respondents were also asked if they experienced any of the following physical illnesses over the last 12 months: heart attack, angina pectoris, cerebral stroke/brain haemorrhage, asthma, hay fever, chronic bronchitis, emphysema, diabetes, osteoporosis, fibromyalgia, severe skin disease, cancer, or severe infection. Those who reported illnesses were coded as having a ‘physical illness’*.* We also tabulated the frequencies of cardiovascular disorders (heart attack/angina pectoris/cerebral stroke/brain haemorrhage); respiratory disorders (asthma/hay fever/chronic bronchitis/emphysema); diabetes; osteoporosis/fibromyalgia; and other illnesses (severe skin disease/cancer/severe infection). Those who did not report any of the listed illnesses were used as the reference group.

The question on pain was: *Have you during the last year suffered from pain and/or stiffness in muscles and joints that have lasted continuously for at least 3 months (Response options: yes/no).*

The question on injuries was: *Have you been injured in service or at work over the last 12 months? (Response options: injury to the muscles/joints/bones/hearing/frostbite/other)*. Those who reported any of the listed injuries were coded as having ‘injury’; those who did not report any of the listed injuries were used as the reference group.

Information was collected on the frequency of use of six drug types: over-the-counter painkillers; prescribed painkillers; sleep medicines; tranquilizers; anti-depressants; and other prescribed drugs in the last 4 weeks. Response options were *daily* (4)*/every week but not daily* (3)*/not every week* (2)*/did not use it* (1)*.* A sum-score of total drug use (range 6–24) was calculated for each participant. We also combined all those who reported using any of the six drug types in the last 4 weeks into a category ‘used any drug’. Finally, we tabulated the frequencies of use of non-prescribed analgesics; prescribed analgesics; psychotropic (medicines for sleep/tranquilizers/depression); and other prescribed drugs separately. Those who had not used any drugs in the last 4 weeks were used as the reference category.

Body mass index (BMI) was calculated as body weight divided by the square of height (kg/m^2^). BMI was not calculated in 58 persons who had missing or extreme values (most likely erroneously registrations) on body weight and/or height. These outliers were defined as z-scores ±3.29 [25]. Obesity was defined as a BMI ≥ 30 [26].

#### Mental health

Mental distress was measured by a mental health index widely used in health surveys in Norway. It includes seven questions on various aspects of mental distress that are partly modified from the General Health Questionnaire [27] and the Hopkins Symptom Check List [28]. The seven questions are: *Have you, in the last 2 weeks, felt; 1) nervous and restless; 2) troubled by anxiety; 3) confident and calm; 4) irritable; 5) happy and optimistic; 6) down/ depressed; or 7) lonely?* Each item has four response options: *no* (1)*/a little* (2)*/moderately* (3)*/very much* (4). Values on items 3 and 5 were reversed in the analysis. Previous research found a Cronbach alpha of .81, and recommended that the index be used as a continuous scale representing different degrees of symptom severity, or as a categorical measure for mental health problems. For the latter, the suggested and commonly used cut-off value is a mean score ≥ 2.15 [29].

The question on mental health treatment was: *Have you over the last 12 months suffered from mental health problems for which you sought help? (Response options: yes/no).*

Post-traumatic stress was measured by the six-item version of the Post Traumatic Stress Disorder Checklist – Civilian version (PCL-C), which consists of items 1, 4, 7, 10, 14 and 15 of the full PCL-C. Respondents were asked to rate the degree to which they were bothered by symptoms related to a stressful experience in the past month on a 1–5 scale, thus total scores ranged from 6 to 30. An individual was considered to have post-traumatic stress disorder (PTSD) if the sum of the items was ≥14 [30–32].

#### Health behaviour

Two questions captured heavy and light leisure time PA, respectively. The questions were formulated in the same way: *How has your physical activity during leisure time been during this last year? Think of your weekly average for the year. Time spent going to work counts as leisure time.”* Both questions had four response options, ranging from *none* (1) to *3 h or more* (4)*.* The sum of these two questions was used as a continuous scale, and was categorised as ‘heavy leisure time PA’ if respondents reported ≥6 h of PA per week. Those who reported < 6 h of PA per week were used as the reference group [33].

The survey included questions on the frequency and amount of *daily smoking, daily use of smokeless tobacco (Swedish “snuff”), and alcohol consumption.* Those who reported consuming > 2 (females) or > 4 (males) alcoholic beverages more than 2–3 times a week were classified as having “high alcohol consumption”; those who consumed fewer alcoholic beverages were included in the reference group [34].

### Statistical analyses

Descriptive statistics were used to calculate the distribution of responses on physical and mental health outcomes for military women, military men, and civilian women respectively. For each dichotomous variable, Pearson’s chi-square test was used to compare differences in responses between the three groups. Univariate logistic regression analysis, with military women as the reference group, was used to estimate the effect of being a military woman compared to being a military man or a civilian woman. For continuous variables (drug use, BMI, mental distress, post-traumatic stress, and leisure time PA) differences in means between military women and military men, and between military women and civilian women, were examined using independent sample’s t-tests.

Effect sizes were calculated in terms of Hedges’ *g (g)* for continuous variables. According to Cohen’s suggestions values of *g* = 0.30 were considered small, *g* = 0.50 medium, and *g* = 0.80 large [35]. For odds ratios (OR), the corresponding values and labels for effect sizes were: OR = 1.68 (small effect), OR = 3.47 (medium effect), and OR = 6.71 (large effect). The equivalent values for OR less than 1.00 would be 0.59 (small effect), 0.29 (medium effect), and 0.15 (large effect) [36].

The participants were divided into three age groups: 20–29 years; 30–39 years; and 40–60 years. This distinction matches Levinson’s theory of social stages in adult life, and empirical work on peak ages for biological capacities and physical performance [37, 38]. We repeated the descriptive analyses in each age-stratum, with results presented as unadjusted *OR*. Frequency distributions of the health problems in each age stratum are available as additional online material (Additional files 1, 2, 3).

Hierarchical multiple regression with robust standard errors were run to assess the combined effect of increasing age, gender and military status on the following outcome variables: drug use; BMI, mental distress; post-traumatic stress; and leisure time PA. Age was entered as a continuous variable in the first step; then gender (step 2) and military status (military vs. civilian) (step 3) were included. Age was centred and the dependent variables were left un-centred [39]. Interactions between age*gender and age*military status were assessed in the fourth step. Individual interaction effects were explored if step 4 resulted in a significant increase in explained variance (*R*^2^). *P*-values < .05 were regarded as statistically significant in all analyses. The analyses were performed in Stata 14.2, StataCorp LLC, Texas, USA.

## Results

### Self-reported physical and mental health in military women compared to military men

In the total population, there were no differences in in self-reported poor health and physical illness between military women and military men (Table 1), but more military women than military men reported pain in the joints/muscles. Frequencies of injuries at work or in service were similar between the sexes. More military women reported use of non-prescribed analgesics and psychotropics, and more military women had mental health issues than military men. However standardized mean differences on the sum-score for drug use and mental distress were small (*g =* − 0.08 and 0.13). On the other hand, military women were clearly leaner (*g* = 0.89) than military men. The women reported some more leisure time PA and less tobacco use than military men. High alcohol consumption did not differ much between military women and military men.

**Table 1:** Health and Health Behaviours in the Norwegian Armed Forces, Numbers (%) (*N* = 10,249)

	Military women *n* = 1068 (10.4)	Military men *n* = 8100 (79.0)	Civilian women *n* = 1081 (10.6)
Age mean, 37.4 (*SD* 11.7)	32.3 (10.1)	**37.1 (11.7)** ^**1**^	**44.8 (9.7)** ^**1**^
Age median, 36 (IQR 21)	29 (16)	35 (22)	46 (15)
20–29 y, *n* = 3556 (34.7)	538 (50.4)	2931 (36.2)	87 (8.0)
30–39 y, *n* = 2323 (22.7)	262 (24.5)	1825 (22.5)	236 (21.8)
40–60 y, *n* = 4370 (42.6)	268 (25.1)	3344 (41.3)	758 (70.1)
Physical health			
Poor health, *n* = 871 (8.5)	87 (8.2)	625 (7.7)	**159 (14.7)** ^**1**^
Physical illness, *n* = 961 (9.4)	88 (8.2)	677 (8.4)	**196 (18.1)** ^**1**^
Cardiovascular disorders, *n* = 29 (0.3)	2 (0.2)	27 (0.3)	0
Respiratory disorders, *n* = 647 (6.5)	67 (6.4)	475 (6.0)	**105 (10.6)** ^**1**^
Diabetes, *n* = 89 (1.0)	1 (0.1)	**70 (0.9)** ^**2**^	**18 (2.0)** ^**2**^
Osteoporosis/fibromyalgia, *n* = 123 (1.2)	10 (1.0)	48 (0.6)	**65 (6.8)** ^**1**^
Other illnesses, *n* = 122 (1.3)	11 (1.1)	82 (1.1)	**29 (3.2)** ^**2**^
Pain, *n* = 2643 (25.8)	296 (27.7)	**1913 (23.6)** ^**2**^	**434 (40.2)** ^**1**^
Injury, *n* = 2491 (24.3)	284 (26.6)	2034 (25.1)	**173 (16.0)** ^**1**^
Drug use			
Sum-score mean, 7.05 (*SD* 1.93) [*g*]	7.07 (1.68)	**6.92 (1.87)** ^**2**^ **[−0.08]**	**7.96 (2.29)** ^**1**^ **[0.43]**
Used any drug, *n* = 4728 (46.2)	566 (53)	**3415 (42.2)** ^**1**^	**747 (69.2)** ^**1**^
Non-prescribed analgesics, *n* = 3780 (36.9)	469 (43.9)	**2721 (33.6)** ^**1**^	**590 (54.6)** ^**1**^
Prescribed analgesics, *n* = 596 (5.8)	63 (5.9)	388 (4.8)	145 (13.4) ^1^
Psychotropics, *n* = 299 (2.9)	39 (3.7)	**197 (2.4)** ^**2**^	**63 (5.8)** ^**2**^
Other prescribed drugs, *n* = 1633 (16.0)	164 (15.4)	1127 (13.9)	**342 (31.7)** ^**1**^
BMI min-max, 14.3–46.3	17.0–37.6	16.4–39.7	14.3–46.3
BMI mean, 25.66 (SD 3.18) [*g*]	23.42 (2.73)	**25.99 (2.88)** ^**1**^ **[0.89]**	**25.43 (4.52)** ^**1**^ **[0.53]**
Obese, *n* = 894 (8.8)	27 (2.5)	**717 (8.9)** ^**1**^	**150 (13.9)** ^**1**^
Mental health			
Mental distress mean, 10.61 (*SD* 3.01) [*g*]	10.97 (3.39)	**10.56 (2.92)** ^**1**^ **[−0.13]**	**10.63 (3.31)** ^**2**^ **[− 0.10]**
Mental health problems, *n* = 706 (6.9)	105 (9.8)	**511 (6.3)** ^**1**^	90 (8.3)
Mental health treatment, *n* = 241 (2.4)	39 (3.7)	**153 (1.9)** ^**1**^	49 (4.5)
Post-traumatic stress mean, 7.12 (*SD* 2.45) [*g*]	7.33 (2.79)	**7.06 (2.32)** ^**2**^ **[−0.11]**	7.38 (2.98) [0.01]
PTSD, *n* = 340 (3.3)	48 (4.5)	**234 (2.9)** ^**2**^	58 (5.4)
Health behaviour			
Leisure time physical activity			
Mean weekly hours, 6.61 (*SD* 1.35) [*g*]	6.89 (1.22)	**6.63 (1.34)** ^**1**^ **[−0.19]**	**6.17 (1.43)** ^**1**^ **[− 0.54]**
Heavy, *n* = 3363 (32.8)	437 (40.9)	**2722 (33.6)** ^**1**^	**204 (18.9)** ^**1**^
Smoking, *n* = 311 (3.0)	15 (1.4)	**203 (2.5)** ^**2**^	**93 (8.6)** ^**1**^
Smokeless tobacco, *n* = 2618 (25.6)	202 (18.9)	**2351 (29.1)** ^**1**^	**65 (6.0)** ^**1**^
High alcohol consumption, *n* = 344 (3.4)	38 (3.6)	262 (3.2)	51 (4.7)

Reference is military women. Statistically significant differences are highlighted in bold

*SD* Standard deviation, *IQR* interquartile range, *g* Hedges’ g, *BMI* body mass index, *PTSD* post-traumatic stress disorder, *PA* physical activity

^1^*p* = < .001 ^2^ *p* < .05

**Fig. 1:**
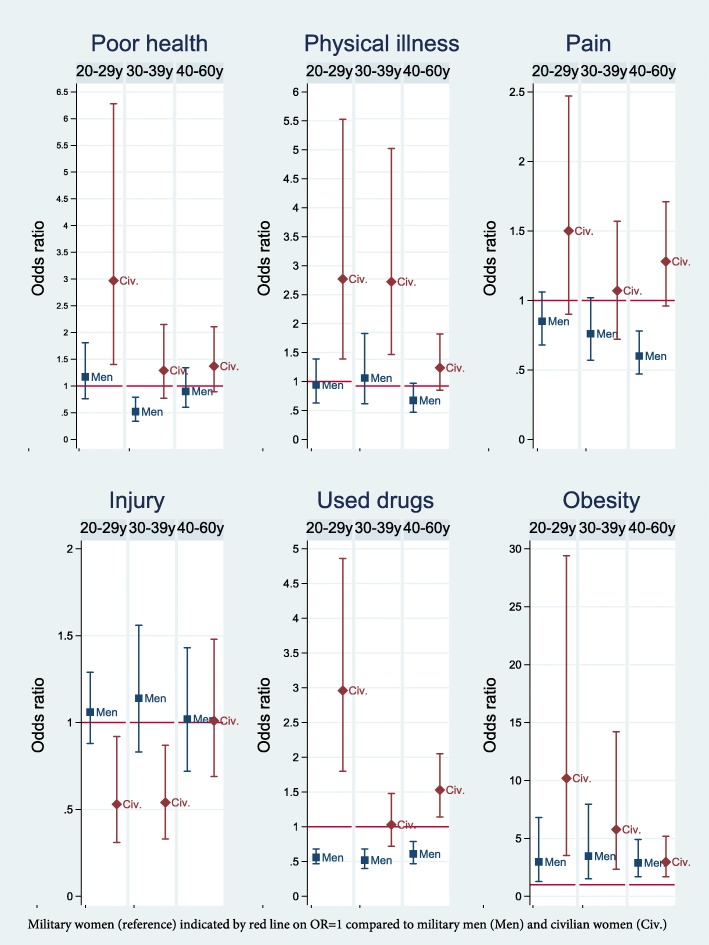
Unadjusted Odds Ratios with 95% Confidence Intervals of Physical health problems in the Norwegian Armed Forces

**Fig. 2:**
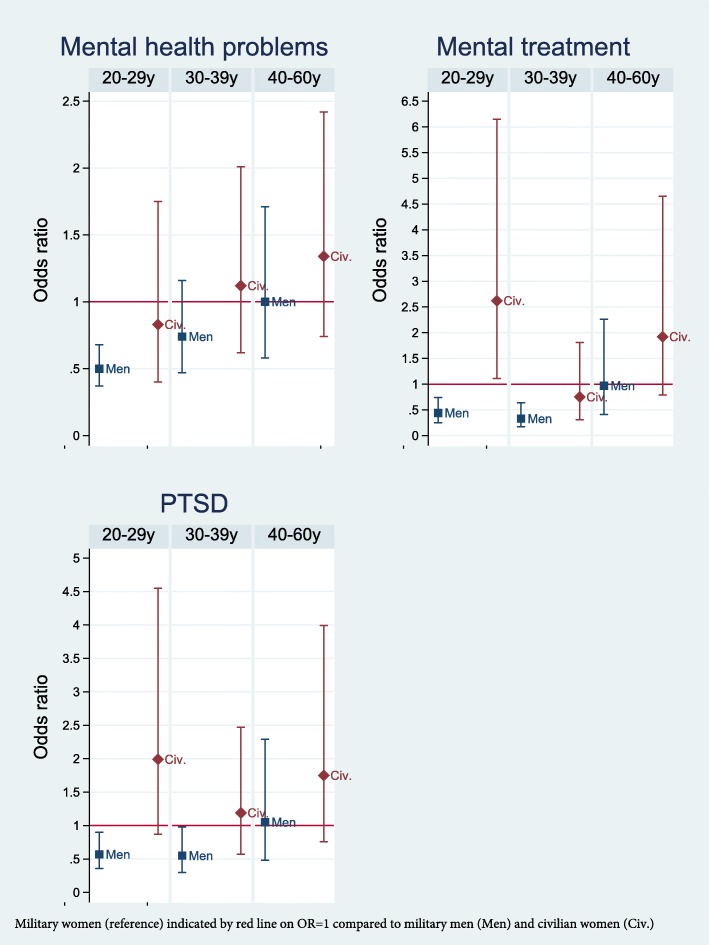
Unadjusted Odds Ratios with 95% Confidence Intervals of Mental health problems in in the Norwegian Armed Forces

**Fig. 3:**
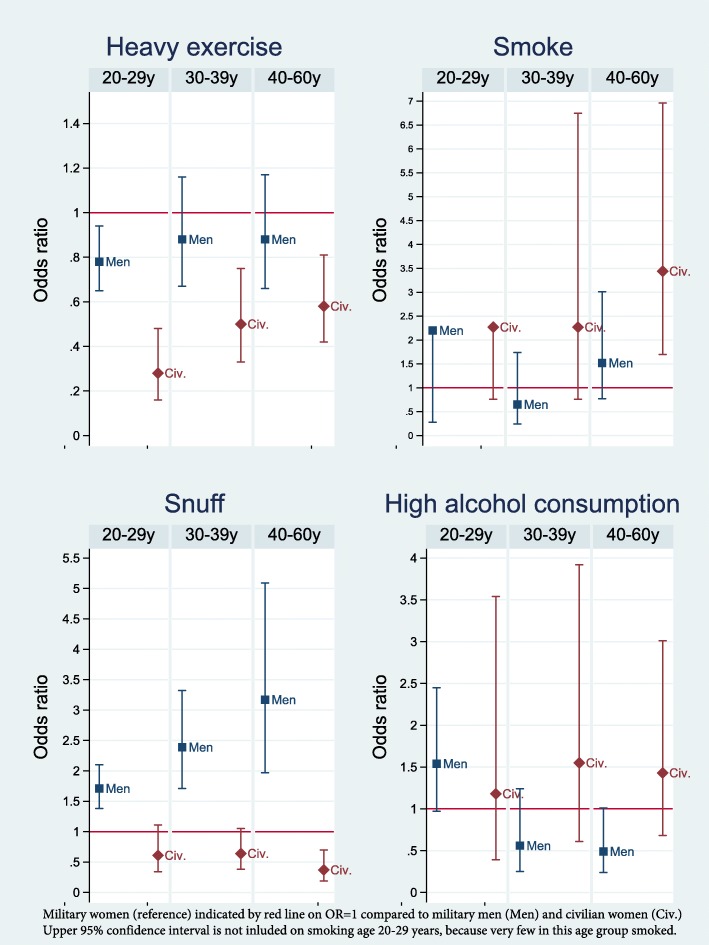
Unadjusted Odds Ratios with 95% Confidence Intervals of Health behavioural problems in the Norwegian Armed Forces

In age-stratified analyses, nearly twice as many military women aged 30–39 years perceived their health as poor compared to military men of the same age (Fig. 1). Few differences in physical illness were present, but among those aged 40–60 years, more military women reported respiratory disorders (11.3% in military women versus 7.1% in military men) and osteoporosis (3% in military women versus 1.3% in military men) [See Additional files 1, 2, and 3]. The finding that more military women reported pain in the muscles/joints compared to military men was statistically significant only in those aged 40–60 years (Fig. 1). In this oldest age group, there were no gender differences in mental health measurements (Fig. 2). A small gender difference in leisure time exercise was statistically significant only in the age group 20–29 years (*g = − 0.13)* (Fig. 3).

### Self-reported physical and mental health in military women compared to civilian women

Overall, military women perceived their health as poor less often than civilian women. They also were less likely to report physical illness, pain in the joints/muscles, drug use, or obesity. However, more military women reported work-related injuries. Mental health measurements did not differ much between military and civilian women. Military women’s reports of leisure time exercise PA surpassed those of civilian women (*g* = 0.54). Military women reported smoking less often than civilian women, but more military women used smokeless tobacco. There was no significant difference in high alcohol consumption between the groups.

In age-stratified analyses, many of the differences between military and civilian women disappeared or levelled out at ages 30–39 and 40–60 years. Still, in each age group there were indications of less drug use among military women; military women were clearly less often obese; and they had a higher level of leisure time PA. Differences between military and civilian women in smoking and smokeless tobacco use were only present in those aged 40–60 years.

### The combined effects of increasing age, gender, and military status on self-reported health

Age (step 1) was an independent predictor of more drug-use, increasing BMI, less mental distress, and less physical activity, but was not associated with symptoms of post-traumatic stress. More specifically, a higher age was associated with more drug use and a higher BMI, but with less mental distress and physical activity.

Gender (step 2) added statistically significantly to the prediction of each outcome and being female was a statistically significant predictor of more drug use, lower BMI, more symptoms of mental distress, more post-traumatic stress, and more leisure time PA. Military status (step 3) added statistically significantly, but explained less than 1% of the prediction of less drug use, lower BMI and more leisure time PA.

**Fig. 4:**
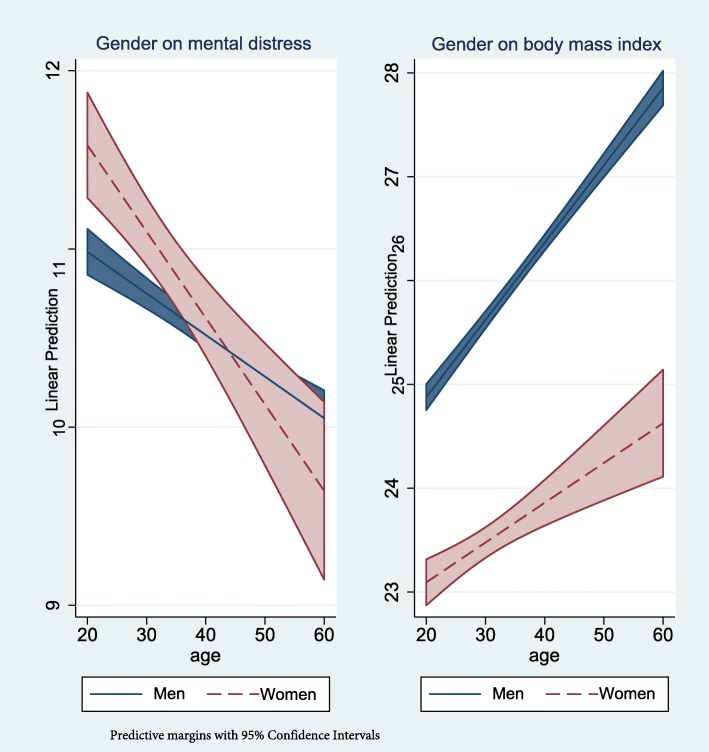
Interactions of Age*Gender on Mental Distress and Body Mass Index in the Norwegian Armed Forces

The three variables age, female gender and military status together added statistically significantly to the prediction of drug use (*F(3, 10,237) = 128.4, p < .001, R*^*2*^ *= .05)*, BMI (*F(3, 10,187) = 518.9, p < .001, R*^*2*^ *= .12)*, mental distress (*F(3, 10,238) = 42.6, p < .001, R*^*2*^ *= .01)*, post-traumatic stress (*F(3, 10,238) = 7.1, p < .001, R*^*2*^ *= .003),* and leisure time PA (*F(3, 10,238) = 192,9, p < .001, R*^*2*^ *= .05).* But, entering the interaction terms into the model (step 4) altered some of the associations to non-significant predictors. Step 4 (interactions) was statistically significant on BMI, mental distress and PTSD (Table 2). We found statistical interaction between age and gender for BMI and symptoms of mental distress (Fig. 4) and no statistical interactions between age and military status.

**Table 2:** Hierarchical multiple regression on health in the Norwegian Armed Forces, *N* = 10,249

	Drug use		Body mass index		Mental distress		Post-traumatic stress		Physical activity	
Factor	Δ*R*^2^	*b*. (*SE*)	Δ*R*^2^	*b*. (SE)	Δ*R*^2^	*b*. (SE)	Δ*R*^2^	*b*. (SE)	ΔR^2^	*b*. (SE)
Step 1: Age	.02^*^	.01 (.009) ^*^	.07^*^	.08 (.01) ^*^	.01^*^	−.01 (.01)	<.001	−.009 (.01)	.04^*^	−.18 (.005) ^*^
Step 2: Female gender	.01^*^	.07 (.19)	.04^*^	−1.06 (.30) ^*^	.001^*^	1.1 (.35) ^*^	.002^*^	.71 (.27) ^*^	<.001^*^	.16 (.14)
Step 3: Military status	.004^*^	−.71 (.40)	.006^*^	−1.00 (.68)	<.001	.09 (.60)	<.001	−.61 (.54)	.005^*^	.65 (.23) ^*^
Step 4: Interactions	<.001		.002^*^		<.001^*^		<.001^*^		<.001	
Gender^*^age		.006 (.005)		−.03 (.009) ^*^		−.02 (.01) ^*^		−.01 (.007)		−.0003 (.004)
Military status^*^age		.004 (.009)		−.01 (.01)		−.008 (.01)		.008 (.01)		−.005 (.005)
Total *R*^2^	.04		.12		.01		.003		.05	

All beta coefficients (unstandardized) were from the full model with all steps included (robust SE)

*SE* standard error

^*^p < .05

## Discussion

This study examined self-reported physical and mental health in active duty military women and compared it to self-reported physical and mental health in active duty military men and civilian women. We expected to find equal levels of physical and mental health between the genders, and fewer health problems in military women compared to civilian women.

Most of the military women in our study reported physical symptoms equal to those of military men. Still, there were some differences between the genders in drug use. Furthermore, we found that in distinct age groups, military women reported different health problems relative to their male counterparts; i.e., more mental health issues in the youngest age group (20–29 years) and more musculoskeletal pain in the oldest (40–60 years). The military women used less smokeless tobacco across all age groups than military men. Military women appeared to be more physically healthy than civilian women in every age group. But only the youngest military women had a better perception of their current health status than civilians, and there were few differences in mental health between these two groups of women.

Gender equality in self-perceived poor health was previously found in a study of the UK military [20]. This, and equal reports of physical illness between military women and military men could be attributed to the military medical selection and training, which works equally for both genders. However, in the general population, working women’s perception of having poorer health relative to men tends to attenuate or even disappear from young to older age, perhaps because the balance between work and family becomes less demanding [7]. Whether this is true for military women has not yet been fully determined [40, 41]. Still, it is tempting to speculate on whether our observation of poorer self-perceived health among military women compared to military men aged 30–39 years could be related to pregnancies and child births in this age [42, 43], which would be important to investigate in future studies.

Military women have been reported to be at higher risk for osteoarthritis, fibromyalgia, and chronic pain [44, 45]. These conditions were little reported in our study, but the well-known excess of musculoskeletal pain among women [9] was present among the oldest participants in our study. Physical activity plays a key role in the prevention of this problem [18]; on the other hand, physical overload and injuries have been suggested as associated risk factors [44]. Given the frequency of musculoskeletal pain and the functional disability associated with such pain, data that can be used to assess whether heavy PA and injuries influence musculoskeletal pain in military women as they age would be of particular interest.

The small gender differences that we found in mental problems and in the use of pain-relieving and psychotropic drugs were interesting, because the genders were equally selected and trained to tolerate stress. Due to the military health requirements, active duty military personnel are not allowed to use drugs for chronic medical conditions or mental health issues [46]. Many studies have concluded that military women have either equal risk or are somewhat more likely to suffer from mental health problems than military men [22]. Some authors have suggested that military women may be subjected to higher job demands, more sexual harassment, and fewer opportunities to express physical or emotional pain than their male peers [47, 48]. If so, our findings could indicate that gender differences in exposure to psychosocial hazards are most pronounced in the initial stages of one’s military career [49, 50]. Our study did not include painful issues that are more common in women such as headaches or menstrual cramps, and we did not have information on the reasons for taking drugs. Thus, we can only speculate as to whether the observed differences in drug use were due to more physical or mental pain in military women [45, 48, 51] or to gender differences in the management of pain [52, 53]. Such issues would be important to elucidate in future studies.

The gender equality in service-related injuries that we observed in our study is contradictory to previous military research [54, 55]. We did not have the data to conclude whether the two genders had distinct positions or were assigned to different tasks and operations in the military, which could influence service-related injuries. Furthermore, we combined several types of injuries into a broad and heterogeneous variable, potentially masking gender differences in injuries at certain anatomic locations [54, 56]. However, inconsistent results on the gender-specific risks of sustaining an injury in the military could reflect crucial differences between nations in military selection, training, equipment, health care, or in symptom reporting [57–59].

The military is often regarded as an occupation during which young people start to use tobacco [60]. In the general population, smokeless tobacco use (Swedish “snuff”) is more common in men [61], and our findings reflected this pattern. Overall, our study showed that, compared to civilian women, fewer military women smoked but more military women used snuff. However, in age-stratified analyses these differences were only present in the oldest age group. We believe this reflects the national smoke-free policy that has been implemented in Norway, and the steadily increasing trend of snuff use among the young [62].

Because military people usually conduct military training and service when they are 20–30 years of age, it was not surprising that more military women in this age group had good self-perceived general and physical health compared to civilians. Some studies found that military women were more fit and had better physical health than civilian women in the general population [17, 21, 63], but we are not aware of comparable research that has investigated the healthy soldier effect in women of different ages on outcomes such as drug use, obesity, or PA habits.

### Strengths and limitations

Among the main strengths of this study is the design, which facilitated comparisons with civilian surveys. It is possible to link these survey data to the comprehensive national health registers in Norway, which provides an excellent opportunity to track military women’s health and drug use as they age and to examine whether any of the self-reported factors in this survey are associated with physician-assessed medical outcomes. The study had a large sample size and an adequate participation rate [64]. Information about military status, sex, and age was retrieved from the Norwegian Armed Forces Personnel database with high quality. The questions used in this survey have been found to be reasonable instruments for measuring health [29, 65]. However, the measurements cannot be used as a surrogate for clinical health assessment or to interpret functional disability. Other important limitations need to be considered when interpreting the results from our study. We cannot rule out differential selection or information bias between military women and men [66, 67]. Measures such as BMI may not be adequate for comparison between genders, because BMI is strongly influenced by muscle mass. Stratification into smaller age groups may have deflated true differences between the personnel groups. Age stratification could not rule out residual confounding within the strata. Particularly in the youngest and the oldest age groups, lower mean age in military women than in civilian women potentially threatened the internal validity of our findings. Linear regression on sum-scores added to the findings from bi-variate analyses in age strata and compensate for the cost of dichotomizing variables [68]. Still, the cross-sectional design hinders causal associations and limits the interpretations of increasing age on health. The women in our study represented three generations of women who may have been subjected to different military selection. Furthermore, the women grew up with dissimilar norms and attitudes towards a range of things that could influence their self-reported health, such as attitudes towards women in the military, mental illness, and pain expression. The study lacked information about relevant biological factors, such as the women’s hormonal and reproductive status. Other important information such as service type, veteran status or rank was not available to the study which limits the interpretation of the multivariate analyses. The results from this study are only indicative and must be interpreted with caution.

## Conclusion

The military women in our study reported physical illness and injuries with the same frequency as military men of similar ages. Nevertheless, more military women reported drug use. In different age groups, military women had different health problems relative to their male colleagues. Military women used drugs less often and were leaner and more physically active than civilian women employed in the Armed Forces. But the reporting of more favourable health compared to civilian women was most evident in the youngest age group and did not apply to mental health outcomes.

## Supplementary information

**Additional file 1. **Health Problems and Behaviour in the Norwegian Armed Forces Age 20–29 years, (*n* = 3556).

**Additional file 2. **Health Problems and Behaviour in the Norwegian Armed Forces Age 30–39 years, (*n* = 2323).

**Additional file 3. **Health Problems and Behaviour in the Norwegian Armed Forces Age 40–60 years, (*n* = 4370).

## Data Availability

The data that support the findings of this study are available from The Norwegian Armed Forces Health Registry but restrictions apply to the availability of these data, which were used under license for the current study, and so are not publicly available. Data are however available from the authors upon reasonable request and with permission of The Norwegian Armed Forces Health Registry.

## Abbreviations

BMI: Body Mass Index
*g*: Hedges’ g
OR: Odds ratio
PA: Physical activity
PTSD: Post Traumatic Stress Disorder
