# Supplementary material for: Fit for fight – self-reported health in military women: a cross-sectional study
Source: BMC Womens Health. 2019 Oct 17;19:119. doi: 10.1186/s12905-019-0820-4 (PMC6798407; doi:10.1186/s12905-019-0820-4)
Supplement: Supplementary file 1 — Additional file 1. Health Problems and Behaviour in the Norwegian Armed Forces Age 20–29 years, (n = 3556). [file 12905_2019_820_MOESM1_ESM.docx]

| **Additional file 1: Health Problems and Behaviour in the Norwegian Armed Forces Age 20-29 years, (*n* = 3,556)** | | | | |
| --- | --- | --- | --- | --- |
|  | **Military women**  **(*n* = 538)** | **Military men**  **(*n* = 2931)** | **Civilian women**  **(*n* = 87)** | |
| Age mean (*SD*) | 24.18 (2.46) | **24.93 (2.50) ^1^** | **26.57 (2.36) ^1^** | |
| Age median (*IQR*) | 24 (4) | 25 (4) | 28 (4) | |
|  |  |  |  | |
| **Physical health** |  |  |  | |
| Poor health | 25 (4.6) | 159 (5.4) | **11 (12.6) ^2^** | |
| Physical illness | 32 (5.8) | 165 (5.6) | **13 (14.9) ^2^** | |
| Cardiovascular disorders | 2 (0.4) | 2 (0.1) | 0 | |
| Respiratory disorders | 28 (5.2) | 142 (4.9) | **13 (14.9) ^1^** | |
| Diabetes | 0 | 1 | 1 (1.3) | |
| Osteoporosis/fibromyalgia | 0 | 8 (0.3) | 0 | |
| Other illnesses | 2 (0.4) | 13 (0.5) | 0 | |
| Pain | 119 (22.1) | 570 (19.5) | 26 (29.9) | |
| Injury | 184 (34.2) | 1047 (35.7) | **19 (21.8) ^2^** | |
| Drug use |  |  |  | |
| Sum score mean (*SD*) [*g*] | 6.83 (1.52) | 6.71 (2.23) [-0.05] | **8.0 (2.46) ^1^ [0.69]** | |
| Used any drugs | 245 (45.5) | **944 (32.2) ^1^** | **62 (71.3) ^1^** | |
| Non-prescribed analgesics | 203 (37.7) | **838 (28.6) ^1^** | **53 (60.9) ^1^** | |
| Prescribed analgesics | 20 (3.7) | 119 (4.1) | **14 (16.1) ^1^** | |
| Psychotropics | 13 (2.4) | 68 (2.3) | **6 (6.9) ^2^** | |
| Other prescribed drugs | 66 (12.3) | **195 (6.7) ^1^** | **24 (27.6) ^1^** | |
| BMI mean (*SD*) [*g*] | 23.15 (2.29) | **25.03 (2.41) ^1^ [0.78]** | **24.35 (4.03) ^1^ [0.46]** | |
| Obesity | 6 (1.1) | **95 (3.3) ^2^** | **9 (10.3) ^1^** | |
|  |  |  |  | |
| **Mental health** |  |  |  | |
| Mental distress mean (*SD*) [*g*] | 11.36 (3.45) | **10.77 (2.76) ^1^ [-0.20]** | 11.38 (3.53) [0.004] | |
| Mental health problems | 65 (12.1) | **191 (6.5) ^1^** | 9 (10.3) | |
| Mental health treatment | 20 (3.7) | **49 (1.7) ^2^** | **8 (9.2) ^2^** | |
| Post-traumatic stress mean (*SD*) [*g*] | 7.39 (2.86) | **7.03 (2.21) ^2^ [ -0.15]** | | **8.07 (3.63) ^2^ [0.22]** |
| PTSD | 26 (4.8) | **83 (2.8) ^2^** | 8 (9.2) | |
|  |  |  |  | |
| **Health behaviour** |  |  |  | |
| Leisure time PA |  |  |  | |
| Mean weekly hours (*SD*) [*g*] | 7.13 (1.13) | **6.97 (1.21) ^2^ [-0.13]** | **6.53 (1.28) ^1^ [-0.52]** | |
| Heavy | 276 (51.3) | **1327 (45.3) ^2^** | **20 (23.0) ^1^** | |
| Smoking | 1 (0.2) | 12 (0.4) | **2 (2.3) ^2^** | |
| Smokeless tobacco | 136 (25.3) | **1074 (36.7) ^1^** | 15 (17.2) | |
| High alcohol consumption | 21 (3.9) | 173 (5.9) | 4 (4.6) | |
|  |  |  |  | |

*Note*. Numbers (%), ^1^p =< .001 ^2^ p < .05. Statistically significant results indicated in bold, reference is military women. Abbreviations: SD=standard deviation, IQR=interquartile range, *g* = Hedge’s g, BMI=body mass index, PTSD=post-traumatic stress disorder, PA=physical activity.
