# Supplementary material for: Fit for fight – self-reported health in military women: a cross-sectional study
Source: BMC Womens Health. 2019 Oct 17;19:119. doi: 10.1186/s12905-019-0820-4 (PMC6798407; doi:10.1186/s12905-019-0820-4)
Supplement: Supplementary file 2 — Additional file 2. Health Problems and Behaviour in the Norwegian Armed Forces Age 30–39 years, (n = 2323). [file 12905_2019_820_MOESM2_ESM.docx]

| **Additional file 2: Health Problems and Behaviour in the Norwegian Armed Forces Age 30-39 years, (*n* = 2,323)** | | | |
| --- | --- | --- | --- |
|  | **Military women**  **(*n* = 262)** | **Military men**  **(*n* = 1825)** | **Civilian women**  **(*n* = 236)** |
| Age mean (*SD*) | 33.74 (2.95) | 34.01 (2.95) | **34.88 (2.69) ^1^** |
| Age median (*IQR*) | 33 (5) | 34 (6) | 35 (4) |
| Lives with spouse/partner | 201 (93.9) | 1438 (95.2) | 176 (93.1) |
|  |  |  |  |
| **Physical health** |  |  |  |
| Poor health | 32 (12.2) | **124 (6.8) ^2^** | 36 (15.3) |
| Physical illness | 16 (6.1) | 128 (7.0) | **38 (16.1) ^1^** |
| Cardiovascular disorders | 0 | 5 (0.3) | 0 |
| Respiratory disorders | 10 (3.9) | 108 (6.0) | **26 (11.6) ^2^** |
| Diabetes | 0 | 3 (0.2) | 1 (0.5) |
| Osteoporosis/fibromyalgia | 3 (1.2) | 0 | 5 (2.5) |
| Other illnesses | 4 (1.6) | 13 (0.8) | 8 (3.9) |
| Pain | 74 (28.2) | 422 (23.1) | 70 (29.7) |
| Injury | 57 (21.8) | 440 (24.1) | **31 (13.1) ^2^** |
| Drug use |  |  |  |
| Sum score mean (*SD*) [*g*] | 7.14 (1.71) | **6.70 (1.20) ^1^ [-0.34]** | **7.48 (2.24) ^2^ [0.17]** |
| Used any drugs | 153 (58.4) | **778 (42.7) ^2^** | 140 (59.3) |
| Non-prescribed analgesics | 137 (52.3) | **693 (38.0) ^1^** | 122 (51.7) |
| Prescribed analgesics | 20 (7.3) | **68 (3.7) ^2^** | 19 (8.1) |
| Psychotropics | 12 (4.6) | **31 (1.7) ^2^** | 11 (4.7) |
| Other prescribed drugs | 30 (11.5) | 150 (8.2) | **46 (19.5) ^2^** |
| BMI mean (*SD*) [*g*] | 23.35 (2.73) | **25.91 (2.74) ^1^ [0.93]** | **24.92 (4.52) ^1^ [0.42]** |
| Obesity | 6 (2.3) | **137 (7.5) ^2^** | **28 (11.9) ^1^** |
|  |  |  |  |
| **Mental health** |  |  |  |
| Mental distress mean (*SD*) [*g*] | 11.03 (3.53) | 10.79 (3.05) [-0.07] | 11.12 (3.49) [0.02] |
| Mental health problems | 25 (9.5) | 133 (7.3) | 25 (10.6) |
| Mental health treatment | 13 (5.0) | **31 (1.7) ^1^** | 9 (3.8) |
| Post-traumatic stress mean (*SD*) [*g*] | 7.52 (3.24) | **7.15 (2.34) ^2^ [-0.14]** | 7.51 (3.12) [-0.0008] |
| PTSD | 15 (5.7) | **59 (3.2) ^2^** | 16 (6.8) |
|  |  |  |  |
| **Health behaviour** |  |  |  |
| Leisure time PA |  |  |  |
| Mean weekly hours (*SD*) [*g*] | 6.71 (1.20) | 6.57 (1.37) [-0.10] | **6.31 (1.33) ^1^ [-0.32]** |
| Heavy | 88 (33.6) | 564 (30.9) | **48 (20.3) ^1^** |
| Smoking | 5 (1.9) | 23 (1.3) | 10 (4.2) |
| Smokeless tobacco | 47 (17.9) | **626 (34.3) ^1^** | 29 (12.3) |
| High alcohol consumption | 8 (3.1) | 32 (1.8) | 11 (4.7) |
|  |  |  |  |

*Note*. Numbers (%), ^1^p =< .001 ^2^ p < .05. Statistically significant results indicated in bold, reference is military women. Abbreviations: SD=standard deviation, IQR=interquartile range, *g* = Hedge’s g, BMI=body mass index, PTSD=post-traumatic stress disorder, PA=physical activity.
