# Supplementary material for: Fit for fight – self-reported health in military women: a cross-sectional study
Source: BMC Womens Health. 2019 Oct 17;19:119. doi: 10.1186/s12905-019-0820-4 (PMC6798407; doi:10.1186/s12905-019-0820-4)
Supplement: Supplementary file 3 — Additional file 3. Health Problems and Behaviour in the Norwegian Armed Forces Age 40–60 years, (n = 4370). [file 12905_2019_820_MOESM3_ESM.docx]

| **Additional file 3: Health Problems and Behaviour in the Norwegian Armed Forces Age 40-60 years, (*n* = 4,370)** | | | |
| --- | --- | --- | --- |
|  | **Military women**  **(*n* = 268)** | **Military men**  **(*n* = 3344)** | **Civilian women**  **(*n* = 758)** |
| Age mean (*SD*) | 47.20 (5.38) | **49.45 (5.60) ^1^** | **50.01 (5.94) ^2^** |
| Age median (*IQR*) | 46.5 (9) | 50 (9) | 50 (10) |
|  |  |  |  |
| **Physical health** |  |  |  |
| Poor health | 30 (11.2) | 342 (10.2) | 112 (14.8) |
| Physical illness | 40 (14.9) | 384 (11.5) | 145 (19.3) |
| Cardiovascular disorders | 0 | 20 (0.7) | 0 |
| Respiratory disorders | 29 (11.3) | **225 (7.1) ^2^** | 66 (9.7) |
| Diabetes | 1 (0.4) | 66 (2.2) | 16 (2.5) |
| Osteoporosis/fibromyalgia | 7 (3.0) | **40 (1.3) ^2^** | **60 (8.9) ^2^** |
| Other illnesses | 5 (2.2) | 56 (1.9) | 21 (3.3) |
| Pain | 103 (38.4) | **921 (27.5) ^1^** | 338 (44.6) |
| Injury | 43 (16.0) | 547 (16.3) | 123 (16.2) |
| Drug use |  |  |  |
| Sum score mean (*SD*) [*g*] | 7.52 (1.87) | **7.23 (1.80) ^2^ [-0.16]** | **8.10 (2.26) ^2^ [0.26]** |
| Used any drugs | 168 (62.7) | **1693 (50.7) ^1^** | **545 (72.0) ^1^** |
| Non-prescribed analgesics | 129 (48.1) | **1190 (35.6) ^1^** | 415 (54.8) |
| Prescribed analgesics | 23 (8.6) | 201 (6.0) | **112 (14.8) ^1^** |
| Psychotropics | 14 (5.2) | **98 (2.9) ^2^** | 46 (6.1) |
| Other prescribed drugs | 68 (25.4) | 782 (23.4) | **272 (35.9) ^2^** |
| BMI mean (*SD*) [*g*] | 24.02 (3.39) | **26.89 (3.07) ^1^ [0.92]** | **25.71 (4.54) ^1^ [0.39]** |
| Obesity | 15 (5.6) | **485 (14.7) ^1^** | **141 (15.0) ^1^** |
|  |  |  |  |
| **Mental health** |  |  |  |
| Mental distress mean (*SD*) [*g*] | 10.11 (2.95) | 10.25 (2.95) [0.04] | 10.39 (3.20) [0.08] |
| Mental health problems | 15 (5.6) | 187 (5.6) | 56 (7.4) |
| Mental health treatment | 6 (2.2) | 73 (2.2) | 32 (4.2) |
| Post-traumatic stress mean (*SD*) [*g*] | 7.04 (2.06) | 7.04 (2.39) [0] | 7.25 (2.84) [0.08] |
| PTSD | 7 (2.6) | 92 (2.8) | 34 (4.5) |
|  |  |  |  |
| **Health behaviour** |  |  |  |
| Leisure time PA |  |  |  |
| Mean weekly hours (*SD*) [*g*] | 6.57 (1.32) | 6.37 (1.37) [-0.15] | **6.08 (1.47) ^1^ [-0.34]** |
| Heavy | 73 (27.2) | 831 (24.9) | **136 (18.0) ^1^** |
| Smoking | 9 (3.4) | 168 (5.0) | **81 (10.7) ^1^** |
| Smokeless tobacco | 19 (7.1) | **651 (19.5) ^1^** | **21 (2.8) ^1^** |
| High alcohol consumption | 9 (3.4) | 57 (1.7) | 36 (4.8) |

*Note*. Numbers (%), ^1^p =< .001 ^2^ p < .05. Statistically significant results indicated in bold, reference is military women. Abbreviations: SD=standard deviation, IQR=interquartile range, *g* = Hedge’s g, BMI=body mass index, PTSD=post-traumatic stress disorder, PA=physical activity.
